# Cues and the optimal timing of activities under environmental changes

**DOI:** 10.1111/j.1461-0248.2011.01686.x

**Published:** 2011-12

**Authors:** John M McNamara, Zoltan Barta, Marcel Klaassen, Silke Bauer

**Affiliations:** 1 School of Mathematics, University of BristolBristol BS8 1TW, UK; 2 Department of Evolutionary Zoology, University of DebrecenDebrecen H-4010, Hungary; 3 Centre for Integrative Ecology, School of Life & Environmental Sciences, Deakin UniversityWaurn Ponds Campus, Geelong VIC 3217, Australia; 4 Department of Animal Ecology, Netherlands Institute of Ecology (NIOO-KNAW)P.O. Box 50, 6700 AB Wageningen, The Netherlands; 5 Swiss Ornithological InstituteSeerose 1, 6204 Sempach, Switzerland

**Keywords:** Cue-response, evolutionary trap, fitness loss, life history activity, migration, mistiming, phenology, photoperiod, reaction norm, reproduction

## Abstract

Organisms time activities by using environmental cues to forecast the future availability of important resources. Presently, there is limited understanding of the relationships between cues and optimal timing, and especially about how this relationship will be affected by environmental changes. We develop a general model to explore the relation between a cue and the optimal timing of an important life history activity. The model quantifies the fitness loss for organisms failing to time behaviours optimally. We decompose the immediate change in fitness resulting from environmental changes into a component that is due to changes in the predictive power of the cue and a component that derives from the mismatch of the old response to the cue to the new environmental conditions. Our results show that consequences may range from negative, neutral to positive and are highly dependent on how cue and optimal timing and their relation are specifically affected by environmental changes.

## Introduction

The timing of important life history activities is widely considered to have significant fitness consequences (e.g. Miller-Rushing *et al.* 2010). Particularly in seasonal environments, timing is the all-dominant predictor of success – for example, timing of growth or reproduction should coincide with favourable conditions and timing of hibernation or dormancy should coincide with unfavourable periods. To make such activities a successful endeavour, they need to be initiated within a specific – often very restricted – time frame. As these activities typically require some mandatory preceding activities (e.g. nest building) or a preparatory period during which body changes occur [e.g. accumulation of body reserves for migration or hibernation (Kunz *et al.* 1998; Madsen & Klaassen 2006)], the organisms must decide on the life history activities well before their actual performance.

Penalties for not starting preparations at the optimal time may range from slight reductions in reproductive success [e.g. raising fewer offspring or offspring with lower survival prospects (Lepage *et al.* 2000)] to fatal consequences [e.g. mistiming of migration leading to starvation (Newton 2007), onset of metamorphosis at a time of high predation pressure (Relyea 2007)]. Besides immediate penalties there may also be time-lagged consequences [e.g. carry-over effects (Harrison *et al.* 2011)], since current mistiming may bear a cost later in life.

Ideally organisms should time their activities in a fitness-maximising manner. Many activities are under photoperiodic control, allowing them to take place within very narrow time periods within the annual cycle (Samach & Coupland 2000; Goldman *et al.* 2004). Such date accurate timing may not be sufficient in the light of environmental variation. Among years, environmental conditions may vary greatly requiring other or additional cues. Besides photoperiod these may include temperature, availability of food and nest sites and intensity of social interactions (Samach & Coupland 2000; Goldman *et al.* 2004). Thus, many organisms have evolved to rely on one or more correlations between environmental cues and windows of opportunity to time their behaviour optimally (e.g. Visser *et al.* 2010; Bauer *et al.* 2011).

Various global processes result in environmental changes whereby formerly reliable cues may no longer be associated with adaptive timing of behavioural and life history decisions (Körner & Basler 2010). This may have important fitness consequences, however, a quantitative approach relating changes in cue and timing to fitness has largely been missing (Sih *et al.* 2011).

Herein we introduce a general model with which we explore the relation between a cue and the optimal timing of an important life history activity. We show how much fitness is reduced if an organism misses the optimal timing because it used an out-dated cue; specifically investigating how this loss depends on the changed relationship between the cue and the optimal timing and the degree of information the cue can give about this timing. The model thus assists in predicting the immediate consequences of environmental change (Hoffmann & Sgro 2011).

We explore several specific cases of environmental changes that have already occurred or are projected to occur in several climate change scenarios (IPCC 2007): (1) the mean values of cue and optimal time change, (2) the mean values of cue and optimal time change and additionally the regression slope between cue and optimal time changes, (3) the correlation between cue and optimal time changes, and (4) the variance of optimal time changes.

## The relationship between a cue and the optimal timing

The optimal time to perform a life history activity in a given year, T*, depends on the environmental conditions that year. Thus, this time varies across years because of year-to-year environmental variation. As T* is the optimal time set by the environmental conditions, rather than an optimal individual timing decision, we refer to T* as the best possible time in the following.

**Figure 1:**
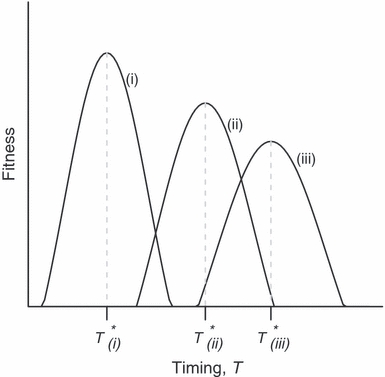
The reproductive value of a hypothetical organism as a function of timing an important life history activity (e.g. breeding), *T*, in three different years (or places); marked by (i), (ii) and (iii) respectively. The peaks of these curves (marked by dashed lines) denote the best possible time in different years (T*_(i)_, T*_(ii)_ and T*_(iii)_ respectively). The penalty for not initiating the activity at the best possible time may differ between years (peaks may be narrower in some year than others) and may depend on the direction of the deviation (curves may be skewed, although they are symmetric in the case illustrated).

**Figure 2:**
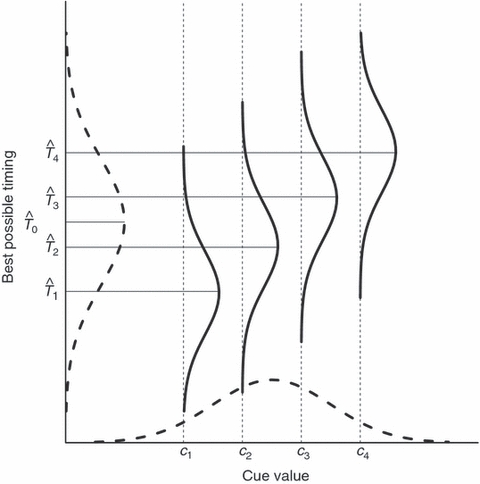
The relation between a cue and the best possible time, T*. The distribution of the cue and the distribution of the best possible time are given next to the corresponding axes (dashed probability distributions). In the absence of any information on the current value of T* the best predicted time 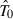 is the mean of T*. A given cue value determines the distribution of T* conditional on this cue. The mean and the variance of this distribution are called the conditional mean and variance. The best predicted time given cue value *c*_*i*_ is 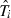, which is the mean of T* given this cue.

The fitness of an organism depends on its own timing, *T*, and the best possible time, T*, in that year (Fig. 1). By sampling over many years, the between-year variation of the best possible time T* can be characterised by a distribution (Fig. 2). Usually when an organism makes decisions on the timing of activities it has limited information on the value of T* for the current year, and must choose the best timing based on the available information. We will refer to this best timing as the best predicted time 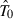. If this life history activity involves preparations, for example, egg laying or incubation, the preparatory activities should be initiated so that the life history activity takes place at this best predicted time.

For clarity of exposition we here make the simplifying assumption that an organism that chooses timing *T* when the best possible time is T* experiences a fitness loss 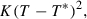
(1) where *K* is a constant (for a generalisation to the cases of variable *K* and skewed costs, see Supporting Information). If assumption (1) holds the best predicted time in the absence of a cue is the mean value of T* averaged across years:


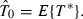
(2)

If there is a cue that gives information about T*, then the best predicted time for a given cue value is the mean of T* averaged for this cue value (Fig. 2). If, for instance, the cue indicating the best possible timing is the date at which a temperature threshold was reached, Fig. 2 should be read as follows: *c*_1_ is a comparatively early date at which this threshold was reached (and similarly, *c*_2_, *c*_3_, *c*_4_ indicate relatively later dates for reaching this threshold). Under *c*_1_, the activity should be performed early. If a cue gave perfect information on the timing of the activity, a single cue value would point out a single value for the best possible time. However, since the cue gives only partial information on timing, the best possible time for the activity varies among years – even at the same cue value – and is therefore distributed around a mean value. Other cue values might include the photoperiod at specific latitudes or the abundance of prey items.

**Figure 3:**
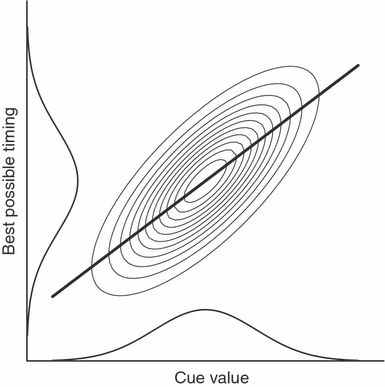
The joint bivariate normal distribution of the best possible time and the cue. The marginal distributions of the best possible time and the cue are plotted next to the appropriate axes. The contour lines illustrate the probability density of the bivariate normal distribution. The heavy straight line is 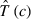 (eqn 3). This line is the least squares regression line of the best possible time T* on the cue *c*, and is also the mean of the best possible time given the cue. It gives the best predicted time given the cue. (The correlation between the cue and the best possible time is *ρ* = 0.75.)

In the further presentation, for simplicity we assume that the joint distribution of T* and the cue is bivariate normal. Figure 3 illustrates contours of the joint probability density for this distribution and also shows the mean of T* given the cue. This straight line is the least squares regression line of T* on the cue. Under the assumption that (1) holds this line is the optimal norm of reaction to the cue.

Let *μ*_*T*_ and 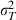 denote the mean and variance of *T* * respectively. Let *μ*_*C*_ and 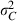 denote the mean and variance of the cue. Finally, let *ρ* denote the correlation between T* and the cue (without loss of generality we assume that the cue is measured in such a way that this correlation is positive). Then the best predicted time given cue value *c* is 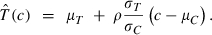
(3)

If an organism does not know the exact value of T* it will typically have a lower fitness value than if it had known T* precisely. We refer to the average fitness reduction as a result of incomplete information as the mean loss, *M*. Equation (3) gives the timing that minimises this mean loss given cue *c* and the mean loss is then *K* times the variance given the cue (Fig. 2). For the bivariate normal distribution the conditional variance is independent of the cue value and the mean loss is 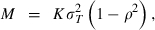
(4) whatever the cue value *c*. Not surprisingly this loss decreases as the correlation between T* and cue increases, that is, the more informative a cue is the better is its predictive power and the lower is the fitness loss.

## Environmental change

Under environmental changes, the best possible timing T* and the cue might change. Changes may affect the mean and/or variance of T* and the cue, and their correlation.

Assuming that the new distribution is again bivariate normal, the new best predicted time given cue value *c* can be expressed in terms of the new parameters (denoted by tildes) as 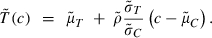
(5)

Similarly, the mean loss as resulting from incomplete information now becomes


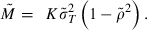
(6)

Before there is adaptive or evolutionary change, population members will continue to use their old response to cues after environmental changes. Assuming these response rules were optimal for the old environment, it can be shown that when cue *c* is received the loss in fitness as a result of the environmental change can be expressed as 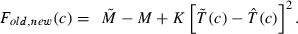
(7)

Two quantities contribute to this overall fitness change. The quantity 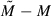 is the fitness consequences of a change in the predictive power of the cue. The quantity 
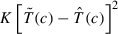
 is the loss due to the fact that the old and new optimal reaction norms differ, and hence represents the extent to which the old reaction norm is not optimal immediately after the change. The average value of *F*_*old*,*new*_(*c*) (averaged over the new cue distribution) will be denoted by 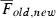.

In the following, we consider various special cases of changes in cue and T* or their relation that are likely to occur under environmental changes, namely changes in (1) the *means* of cue and best possible time, (2) the *means* of cue and best possible time and the *regression slope*, (3) the *correlation* between cue and best possible time, or (4) the *variance* in best possible time.

### Only the means change

**Figure 4:**
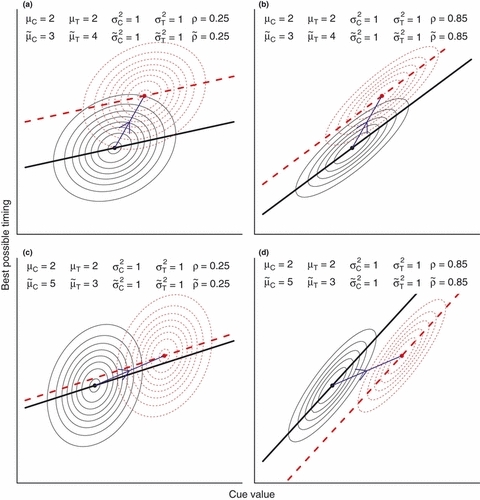
The effects of environmental changes, herein exploring the case that only the means of cue or best possible time change. The plots show the probability densities (contour lines) and the means (heavy dots) of the joint bivariate normal distributions of the best possible time and the cue and the best predicted time (heavy straight lines) before (solid lines) and after (dashed lines) the environmental change. Original parameters (i.e. before cue or timing changed) in all subplots: mean and variance of cue *μ*_*C*_ = 2, 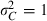, mean and variance of best possible time *μ*_*T*_ = 2, 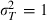 respectively. Four exemplary changes are shown: (a) *α*_*T*_ > *α*_*C*_ and a weak correlation between cue and best possible time, (b) *α*_*T*_ > *α*_*C*_ and a high correlation, (c) *α*_*C*_ > *α*_*T*_ and a weak correlation, (d) *α*_*C*_ > *α*_*T*_ and strong correlation.

When only the means of the cue and/or the best possible time change, eqns (4) and (6) show that 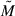
and *M* are equal. In this case the old and new best predicted times are parallel lines and 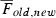 is *K* times the vertical distance between these lines (Fig. 4).

We represent the changes in the means of cue and best possible time in units of their standard deviations and define *α*_*T*_ and *α*_*C*_ by 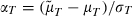 and 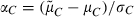 as the relative shifts in these means. Then 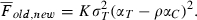
(8)

This quantity is zero if *α*_*T*_ = *ρα*_*C*_, otherwise it is positive, indicating a loss in fitness. If the relative shift in the best possible time is greater than the relative shift in the cue (*α*_*T*_ > *α*_*C*_, Fig. 4a,b), for given *α*_*T*_ and *α*_*C*_, the loss is greatest when the original correlation *ρ* is small. This can be seen by comparing Fig. 4a,b; the vertical distance between the old and new best predicted times is greater in Fig. 4a than that in Fig. 4b. Conversely suppose that the relative shift in the best possible time is less than the relative shift in the cue (*α*_*C*_ > *α*_*T*_, Fig. 4c,d). Then as *ρ* increases, the loss first declines to a minimum of zero when *ρ* = *α*_*T*_/*α*_*C*_, and then increases as *ρ* increases further. In Fig. 4c, the vertical distance between the old and new best predictors is less than in Fig. 4d. Thus, the same environmental changes need not have the same effect but depend very much on the correlation before environmental changes took place, with initially very high or very low correlations being worse than moderate correlations.

### Means and regression slope change

**Figure 5:**
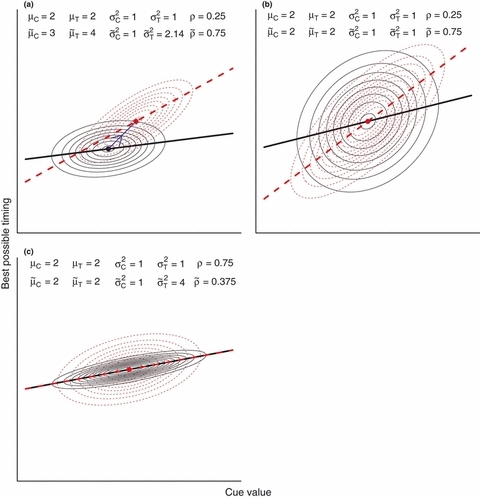
Exploring several special cases of environmental changes: (a) means of cue and best possible time change (*μ*_*T*_ = 2, 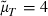, *μ*_*C*_ = 2, 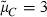) and additionally, the regression slope changes from *ρ* = 0.25 to 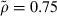 (but errors about the regression line were held constant, 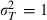, 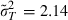, while 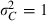), (b) the correlation changes from *ρ* = 0.25 to 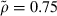, that is, slope of regression and the error about the regression line change and (c) the variance of the best possible time changes (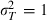, 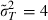) while the means and the regression slope are held constant (*ρ* = 0.75, 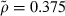). See legend of Fig. 4 for more information.

Suppose that the mean of the cue and/or the best possible time is changing, the slope of the best predicted time also changes, but the errors about this regression line are unchanged (Fig. 5a). [This can be achieved by altering some or all of the parameters *μ*_*C*_, *μ*_*T*_, *α*_*C*_, *α*_*T*_, *ρ* while keeping 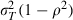 unchanged.] In this case 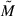
and *M* are again equal. Let Δ_*slope*_ denote the change in slope of the best predicted time. Then it is straightforward to show that 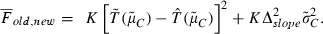
(9)

The two terms on the right hand side of this equation are both positive. Thus, there is fitness loss due to two sources; the first term is *K* times the vertical distance between the old best predicted time and the point 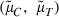 on the new best predicted time, and represents the deleterious effect of changes in the mean of the best predicted time. The second term represents the deleterious effect of the change in the slope of the best predicted time.

### The correlation changes

In the above case the slope of the best predicted time changes, but the error about this regression line is kept constant. Here in contrast we look at the effect of increasing *ρ* holding all other variables constant (Fig. 5b). Increasing *ρ* has two effects; it increases the slope of the best predicted time, so that the old best predictors is not adapted to the new environment, but it also reduces the error about this best predicted time [which is 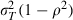]. The net effect of such changes is 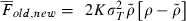. This is negative provided the new correlation 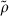 exceeds its old value *ρ*, so that fitness increases. Conversely, decreasing the correlation between cue and the best possible time leads to a loss in fitness.

### The variance in the best possible timing increases

Suppose that the mean of the cue, its variance, and the mean best possible time do not change. Furthermore, suppose that the slope of the best predicted time does not change. Instead the variability in the best possible time increases. (This can be achieved by increasing 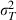 while keeping *ρσ*_*T*_ constant.) In this case the fitness loss increases proportionally to the increase in the variance in the best possible time (Fig. 5c).

## Discussion

Our model conceptualises and characterises the relationship between a cue and the best possible timing of an important life history activity, and quantifies the fitness consequences of changes in their relationship. We also decompose these overall fitness consequences into a component that results from changes in the cue's predictive power and a component that derives from the mismatch of the old best response to the cue and the best possible time under the new environmental conditions.

We assumed that organisms can estimate the best possible time using environmental information. Among the cues identified, photoperiod is probably the most prominent and universal, entraining organisms to time of year. It has been shown to influence many activities in animals (see Bradshaw & Holzapfel 2007 for review) and plants (Jackson 2009; Körner & Basler 2010; Tooke & Battey 2010). Other cues identified include temperatures (e.g. egg laying in birds: Gienapp *et al.* 2005; Visser *et al.* 2009; migratory departure: Bauer *et al.* 2008; Keefer *et al.* 2009; emergence time in insects: Harper & Peckarsky 2006; spawning of corals: Mendes & Woodley 2002), rainfall (e.g. conception in buffalos: Ryan *et al.* 2007; germination: Levine *et al.* 2008; or leaf flushing: Williams *et al.* 2008), moon phase (migratory departure: Meunier *et al.* 2008; spawning of holothurians: Mercier *et al.* 2007) or chemical substances related to presence of natural enemies (e.g. metamorphosis in amphibians: Vonesh & Warkentin 2006; Gomez-Mestre *et al.* 2008; and fish: Wedekind & Muller 2005).

As shown by eqn (4) the predictive power of cues determines fitness loss (in the unchanged case, i.e. before environmental changes took place). A single cue will often only have a low correlation with the best possible time. Dependence on this cue risks severely mistiming important activities and hence risks reproductive failure. Combining multiple cues likely increases the correlation between cue and the best possible time, and thus the cue's predictive power and reliability. Therefore, we hypothesise that for the most sensitive activities in an organism's life history, that is, those activities with the highest fitness consequences, multiple cues should be used. As an example, photoperiod and temperatures have been shown to determine jointly migratory progression in geese, with the weighting given to each factor changing with every stage of the migratory journey (Bauer *et al.* 2008).

Despite the examples given above, our present knowledge of cues remains limited – both with regard to their identity and their quantitative effect on timing (e.g. Bauer *et al.* 2011). This seriously hampers our ability to predict how organisms are affected by (climatic) changes which are likely to affect the cues, the optimal timings of activities and their relation. This situation is further complicated by the fact that the magnitude and direction of these changes depend on the focal species.

Moreover, some cues may not change at all, nor change at the same rate everywhere if they do so. Photoperiod is probably the most prominent example for a cue that has not changed. Whether or not, by how much and in which direction other cues like temperature or rainfall patterns change, cannot be answered without considering the specific environments where they are used.

In Fig. 4, both the cue and the optimal time change while their correlation remains unchanged. This may apply to organisms that use cues such as temperature when future food abundance is determined by temperature. Cues such as vegetation phenology may act similarly (Thomas *et al.* 2010; Studds & Marra 2011; Valtonen *et al.* 2011). Environmental change may affect the optimal timing of activities more than the cue. This will hold where photoperiod is the only cue used (Both *et al.* 2005), for example, in many long-distance migrants that rely on photoperiod for initiating spring migration, and have experienced serious fitness consequences leading to population decline as the optimal arrival time in the (European) breeding grounds has changed (Møller *et al.* 2008; Jones & Cresswell 2010).

Environmental changes in which the cue changes much more than the best possible time (Fig. 4c,d) may also occur frequently, for example, in species higher in the food chain, where a biotic cue indicates optimal timing and is itself affected by environmental changes (Thackeray *et al.* 2010). Here, the deleterious effects of change may be greatest when the correlation between the cue and the best possible time is high.

Environmental changes will not only affect ‘means’ but may also affect the variability in specific events or the cue/best possible time correlation. We have investigated some such changes (Fig. 5).

Man-made habitat changes combined with climate changes could lead to the changes shown in Fig. 5a, where on top of changes in the means of cue and best possible time their correlation changes. If the correlation decreases, the cue loses predictive power, which applies to many habitat alterations, for example, from natural to cultural habitats, disturbances or hunting (Gunnarsson *et al.* 2006; Klaassen *et al.* 2006). This phenomenon has been referred to as an ‘evolutionary trap’ and although mainly applied to habitat choice, it describes any decision (and thus, also a phenological response) that is now maladaptive because of a sudden anthropogenic disruption (Schlaepfer *et al.* 2002).

Although an increased correlation with environmental change (Fig. 5b) may appear unlikely, such effects may occur, for example, the climate-driven changes in the phenology of spruce trees and spruce budworm (*Pseudotsuga menziesii* and *Choristoneura occidentalis* Freeman) that have increased the synchrony between their life-cycles (Miller-Rushing *et al.* 2010), larval butterflies (*Polygonia c-album*) that perform better, that is, grow faster and survive better on host-plants that were newly colonised with climate-driven range expansions (Braschler & Hill 2007) or the laying date of willow tits (*Poecile montanus*) that is now better synchronised with food peaks (Vatka *et al.* 2011).

An increasing variability in timing has also been predicted as a consequence of environmental changes. There is already evidence for detrimental effects of an increased variability in optimal timing that our model predicts (McLaughlin *et al.* 2002) (Fig. 5c).

Many studies from a range of species across many taxa have already shown the demographic consequences of an inadequate response to an altered phenology, for example, population trends of migratory birds were related to whether or not they changed their spring migration timing (Møller *et al.* 2008; Saino *et al.* 2011); non-native plant species were better able to respond compared to native species and thus were highly successful and invasive (Willis *et al.* 2010).

Our model can be extended in several directions. We have assumed that the joint distribution of cue and best possible time is bivariate normal. A consequence is that best predictors are linear in the cue value. Any application of our theory to specific cases would require validating this assumption and scrutinising the consequences if it is violated, particularly if distributions turn out to be long tailed. We have also assumed that the costs of deviation from the best possible time are symmetric. When costs are skewed the best predictor is no longer the mean of the best possible time (see Supporting Information). The effects of environmental change will then be strongly dependent on the direction of change; for example if arriving too early on the breeding grounds is fatal for a migrating bird then an advancement of spring on the breeding grounds may only have mild fitness consequences whereas a delay in spring could be catastrophic.

Besides relaxing some of the assumptions of our model, it could also be extended to, for example, include individual differences as recent empirical work suggests that individuals of different quality respond differently to climate change (Møller 2008), or take into account the mechanistic details of how organisms respond to cues – in a stepwise or a more continuous manner. Another important process that might be considered in future model-extensions is learning, which – dependent on the organisms’ capacities – could potentially alleviate some of the negative consequences of environmental changes (Sih *et al.* 2011).

Unfortunately, at present our knowledge of the proximate factors regulating phenology and the relation between cues and timing is limited, as is the demographic consequences of environmental change on populations, ecosystems and evolutionary processes (Forrest & Miller-Rushing 2010). Nevertheless, our model can generate predictions regarding the severity of changes even when only general (qualitative) information regarding the original and changed relationships is at hand, that is, impressions of the old and new mean optimal timing and cue values, their respective variance, as well as their correlation. It is the changing magnitude of these that determine the fitness changes in the light of environmental change. As outlined above, those changes need not exclusively have negative fitness effects and may vary substantially in their extent.

## SUPPORTING INFORMATION

**Appendix S1** Generalisation to the case of variable *K* and skewed costs.

As a service to our authors and readers, this journal provides supporting information supplied by the authors. Such materials are peer-reviewed and may be re-organized for online delivery, but are not copy-edited or typeset. Technical support issues arising from supporting information (other than missing files) should be addressed to the authors.
