# Supplementary material for: Cues and the optimal timing of activities under environmental changes
Source: Ecol Lett. 2011 Dec;14(12):1183–90. doi: 10.1111/j.1461-0248.2011.01686.x (PMC3258420; doi:10.1111/j.1461-0248.2011.01686.x)
Supplement: Supplementary file 1 [file ele0014-1183-SD1.pdf]

Supporting Information

Appendix S1. Generalisations to the case of variable *K* and skewed costs

In the model, we have assumed that the relation between timing and fitness is given by equation (1). Here we investigate how the best predicted time changes when this assumption is relaxed in two specific ways.

K not constant

In equation (1) the value of *K* does not depend on the value of the best possible time *T**. This means that the width of the peaks in Figure 1 does not depend on *T**. If the width of the peaks are allowed to depend on *T**, *K* is then function of *T** and it can be shown that now

$$\hat{T}_0 = E\{T^*\} + Cov(T^*, K) / E\{K\}. \qquad \text{(S1)}$$

Thus for given $E\{T^*\}$ and $E\{K\}$, the best predicted time increases as the covariance between T^* and *K* increases. This is because it is more important to get the timing right when *K* is large than when it is small.

Costs not symmetric

Since reproductive value reaches a maximum at *T**, small deviations from this timing incur quadratic costs as is assumed by equation (1). In particular the costs of small deviations are symmetric about *T**. However, this need not necessarily be the case for larger deviations as

there could be a higher (lower) penalty for being early than for being late. In other words, the peaks shown in Figure 1 may be skewed. For instance, a migratory bird that arrives too early on Arctic breeding grounds might experience fatal consequences if body reserves are exhausted and foraging is virtually impossible (e.g. because the snow cover is still there), while arriving too late may (only) come at a reduced reproductive output (Drent 2006, Newton 2007). In such cases the best predictor will deviate from the mean of T^* (Fig. 2) even when K is constant.

To investigate this deviation in an especially simple case we assume that the cost associated with the timing T when the best possible time is T^* is of the form $L(T - T^*)$. Here the function L satisfies

$$L'(0) = 0 \tag{S2}$$

and

$$L''(0) > 0. \tag{S3}$$

The best predicted time (in the absence of a cue) satisfies

$$E\{L'(\hat{T}_0 - T^*)\} = 0. \tag{S4}$$

To find an approximate solution of this equation we Taylor expand the function L' about 0 using equation (S2) to give

$$L'(\hat{T}_0 - T^*) = (\hat{T}_0 - T^*)L''(0) + \frac{1}{2}(\hat{T}_0 - T^*)^2 L'''(0). \tag{S5}$$

It is convenient to write $D = \hat{T}_0 - E\{T^*\}$. Using approximation (S5) equation (S4) gives

$$D + \frac{1}{2}\frac{L'''(0)}{L''(0)}\left[D^2 + Var(T^*)\right] = 0 . \qquad \text{(S6)}$$

D can then be found by solving this quadratic equation. When losses are symmetric we have $L'''(0) = 0$, so that equation (S6) predicts that $D = 0$; i.e. $\hat{T}_0 = E\{T^*\}$. In general

$$\hat{T}_0 < E\{T^*\} \quad \Leftrightarrow \quad L'''(0) > 0 . \qquad \text{(S7)}$$

Thus the best predictor is less than the mean of *T** if and only if costs are skewed so that the cost of a given absolute deviation of *T* from *T** is greater when *T* is greater than *T** than when *T* is less than *T**.

References

Drent R.H. (2006). The timing of birds' breeding seasons: the Perrins hypothesis revisited especially for migrants. *Ardea*, 94, 305-322.

Newton I. (2007). Weather-related mass-mortality events in migrants. *Ibis*, 149, 453-467.
